# Genetic confirmation of *Octopus insularis* (Leite and Haimovici, 2008) in South Florida, United States using physical features and *de novo* genome assembly

**DOI:** 10.3389/fphys.2023.1162807

**Published:** 2023-06-20

**Authors:** Brigid Maloney, Eric Angel Ramos, Chelsea O. Bennice, Frank Young, Marcelo O. Magnasco

**Affiliations:** ^1^ Laboratory of Integrative Neuroscience, The Rockefeller University, New York, NY, United States; ^2^ Department of Biological Sciences, Florida Atlantic University, Boca Raton, FL, United States; ^3^ Dynasty Marine, Marathon, FL, United States

**Keywords:** Brazilian reef octopus, *Octopus vulgaris*, Octopus americanus, genomic assembly, cryptic species, cephalopod genetics, *de novo* assembly, body patterns

## Abstract

The distribution of octopuses within the *Octopus vulgaris* species complex remains inadequately understood. Species determination can be complex and involves characterizing a specimen’s physical features and comparing its genetic makeup to other populations. In this study, we present the first genetic confirmation of *Octopus insularis* (Leite and Haimovici, 2008) inhabiting the coastal waters of the Florida Keys, United States. We employed visual observations to identify species-specific body patterns of three wild-caught octopuses and used *de novo* genome assembly to confirm their species. All three specimens exhibited a red/white reticulated pattern on their ventral arm surface. Two specimens displayed body pattern components of deimatic display (white eye encircled by a light ring, with darkening around the eye). All visual observations were consistent with distinguishing features of *O. insularis*. We then compared mitochondrial subunits COI, COIII, and 16S in these specimens across all available annotated octopod sequences, including *Sepia apama* (Hotaling et al., 2021) as a control outgroup taxon. For species exhibiting intraspecific genomic variation, we included multiple sequences from geographically distinct populations. Laboratory specimens consistently clustered into a single taxonomic node with *O. insularis*. These findings confirm *O. insularis* presence in South Florida and suggest a more extensive northern distribution than previously assumed. Whole genome Illumina sequencing of multiple specimens enabled taxonomic identification with well-established DNA barcodes while also generating the first *de novo* full assembly of *O. insularis*. Furthermore, constructing and comparing phylogenetic trees for multiple conserved genes is essential for confirming the presence and delineation of cryptic species in the Caribbean.

## 1 Introduction

The taxonomy of benthic octopuses in the shallow waters of the tropical western Atlantic Ocean, Gulf of Mexico, and Caribbean Sea is complex. Numerous octopus studies focus on characterizing a single species’ life history traits, trophic interactions, or ecological role (Ambrose, 1988; Forsythe and Hanlon, 1988; Aronson, 1989; Anderson et al., 2008; Leite et al., 2009; de Beer and Potts, 2013). However, multiple species coexist in shallow areas and can exhibit similarities in morphology and behavioral phenotypes (Hanlon et al., 2008; O’Brien et al., 2021). Consequently, they can be easily mistaken for the same species or misidentified, leading to inaccurate reports of biodiversity, population size, and food web dynamics. These cryptic octopus species coexist in regions where molecular research has been limited until recently (Norman, 2003).

Octopuses have experienced significant taxonomic revision and expansion, particularly in the western Atlantic Ocean, Gulf of Mexico, and Caribbean regions (Norman, 2003; O’Brien et al., 2021). The genus *Octopus*, once viewed as a “catch-all” genus, has been discovered to be polyphyletic (Guzik et al., 2005), and now encompasses numerous cryptic species—morphologically similar yet genetically distinct octopus species (Knowlton, 1993; Amor et al., 2016). *Octopus vulgaris* (Cuvier, 1797), originally described by Cuvier (1797) from the Mediterranean, was once considered a cosmopolitan species distributed across tropical, subtropical, and temperate waters. Due to geographic and temperature boundaries (while still exhibiting morphologically similar traits), type species names based on location were assigned to *O. vulgaris*: *sensu stricto* (Mediterranean, northeast Atlantic), Type I (western Atlantic), Type II (southwest Atlantic: Brazil), Type III (South Africa and Indian Ocean), and Type IV (east Asia). However, the taxonomic resolution remained elusive. Mitochondrial genes have been used to distinguish *O. vulgaris* species on both coasts of the Americas (COIII and 16s, Sӧller et al., 2000; Warnke et al., 2002; 2002), in the Atlantic Ocean, South Africa, Japan, and Taiwan Province of China (16S and COIII, Warnke et al., 2000), from Brazil (16S, Leite et al., 2008), and from Amsterdam and Saint Paul islands (COI and COIII, Guerra et al., 2010). By integrating molecular and morphological data, studies have been able to identify multiple distinct species Xu et al., 2022; Santana-Cisneros et al., 2021. However, results still indicate the existence of a single, widely distributed *O. vulgaris*.

Advancements in technology, including underwater photography, videography, and morphological and molecular tools, has led to further recent taxonomic revisions, and resulted in the naming, renaming, and redescribing of numerous octopus species throughout western Atlantic and Caribbean (Amor et al., 2016; Guerrero-Kommritz and Camelo-Guarin, 2016; Guerrero-Kommritz and Rodriguez Bermudez, 2019; Avendaño et al., 2020a; O’Brien et al., 2021). As a result, *O. vulgaris* and close relatives have formed the *Octopus vulgaris* species complex including at least six species: *O. vulgaris sensu stricto*, *O. vulgaris* Type III, *Octopus sinensis*, *Octopus tetricus, O. cf. tetricus*, and *O. americanus* (Amor et al., 2016; 2017; 2019; Gleadall, 2016; Avendaño et al., 2020a). Both once described as *O. vulgaris*, *Octopus insularis* (Leite and Mather, 2008) is shown to be morphologically distinct and genetically distant from the complex (although still mentioned as a member of the *O. vulgaris* group) and *O. americanus* (Froriep, 1806) is proposed to be the reinstated name for conspecifics *O. vulgaris* Type I and Type II due to genetically distant results from *O. vulgaris sensu stricto* (Ritschard et al., 2019; Avendaño et al., 2020a; Lima et al., 2020b). These molecular, morphological, and behavioral observations and revisions spanning over two decades highlight the challenges of cryptic species identification and emphasize the need to explore innovative methodologies while optimizing existing phylogenetic techniques for octopuses. As arguably the most intelligent invertebrates, octopuses possess a genome size several times larger than other sequenced molluscan and lophotrochozoan genomes (Albertin et al., 2015; Amor et al., 2019).

*Octopus insularis* was historically believed to inhabit only the tropical waters of reef and rocky substrates off the coast of Brazil. However, recent findings have identified this species in various locations, including the Gulf of Mexico, Turks and Caicos, the Bahamas, Bermuda, and southeastern Florida (Leite et al., 2008; Leite et al., 2009; Sales et al., 2013; Lima et al., 2017; González-Gómez et al., 2018; Rosas-Luis et al., 2019; Lima et al., 2020a; O’Brien et al., 2021; Lima et al., 2023). The presence of *O. insularis* in these newly discovered ranges was determined through underwater photography and videography, identifying species-specific body pattern components and habitat features (Lima et al., 2017; Gonzalez-Gomez et al., 2018; O’Brien et al., 2021). While these methods are useful for investigating species presence in new areas, genetic analysis is crucial for studying cryptic species found in the western Atlantic. Furthermore, understanding both the known and proposed expanded distribution in the region and connecting the identified populations requires genetic analyses of sampled animals (e.g., Ritschard et al., 2019). For example, genetic samples were recently used to confirm *O. insularis* occurrence off the coast of West Africa (Lima et al., 2023).

Genetic tools such as DNA barcodes have become increasingly important for confirming species identity in octopuses. However, to optimize the detection of interspecific differences, previous studies have employed multiple established barcodes to distinguish between species (Hollenbeck et al., 2017; Avendaño et al., 2020a). Sequences used as barcodes typically meet the following criteria: 1) they exhibit significant variation between species; 2) they are flanked by highly conserved regions, allowing the use of universal PCR primers to excise these regions; and 3) they are short regions (less than 1 k bp) that can be easily amplified (Kress and Erickson, 2008). With the advancement of next-generation sequencing technologies, the cost of generating long reads has decreased dramatically, enabling researchers to characterize biodiversity across the animal kingdom in an unprecedented way (Hotaling et al., 2021). Whole genome sequencing is especially important in octopod studies, as they possess expansions of certain subsets of gene families exceeding that of other invertebrates (Albertin et al., 2015).

*De novo* genome assembly, a technique for constructing an entire genome from raw sequencing data of a specimen without referencing a known species’ complete genome, has been employed to reconstruct the genomes of various octopus species Zarrella et al., 2019. This approach offers insights into the genetic differences and evolutionary trajectories of different taxa (Kim et al., 2018; Li et al., 2020). It is particularly valuable for accurately characterizing cryptic complexes, as it minimizes the chance of bias introduced by aligning reads to the reference genome of a closely related taxon. The objective of *de novo* genome assembly is to obtain the most contiguous and accurate representation of the original genome by sequencing millions of DNA fragments (reads) assembling overlapping reads into longer contiguous sequences (contigs). Scaffolds, larger genomic sequences with gaps between contigs, are then constructed by identifying where reads present in the contigs are arranged larger fragments with known length, but undetermined sequence (Waterston et al., 2002; Baker, 2012). Assembly is further refined through error correction and consensus building. By sequencing and assembling the genomes of individuals from different populations, researchers can identify unique genetic signatures specific to each species. These genetic signatures can then be employed to differentiate cryptic species and provide evidence of their distinctiveness. Examining single nucleotide changes across conserved regions allows for an understanding of how evolutionary selection rates vary across the genomes of related species (Wu et al., 2018). In addition to characterizing trends across large genomic regions, shorter barcode regions within these sequences can be compared across species for identification. This approach echoes previous methods by allowing for comparison with all species for which barcodes have been generated.

In this study, we report the presence of *O. insularis* in the Florida Keys, United States, firstly by visual identification (body patterns and components) and secondly by confirmation through genetic analysis. Our research includes a description of the body patterns and components of the sampled animals, and the first use of *de novo* genome assembly for species confirmation of *O. insularis*. We discuss the significance of *O. insularis’* presence in areas bordering the Gulf of Mexico and the western Atlantic Ocean as well as this molecular tool in identifying cryptic cephalopod species.

## 2 Methods

### 2.1 Animal origins and housing

**FIGURE 1:**
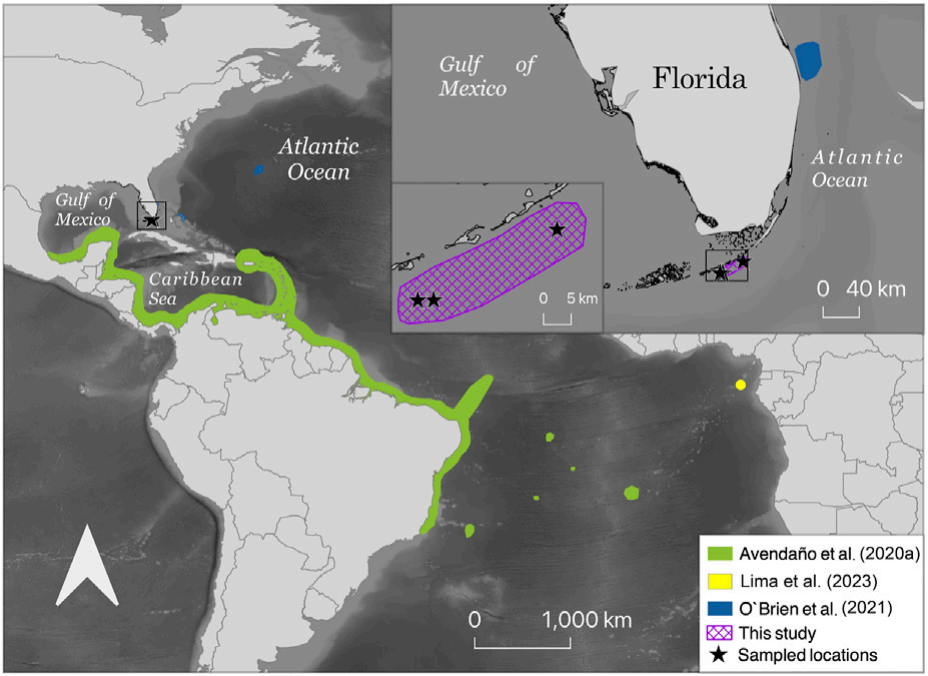
Map of collected *Octopus insularis* in the Florida Keys, Florida, United States, and the proposed expansion of the known range of *O. insularis*. All three octopuses sampled in this study were collected in shallow waters off the Florida Keys where the Gulf of Mexico meets the Atlantic Ocean. The main map shows the distribution range of *O. insularis* from O’Brien et al. (2021); Avendaño et al. (2020a), and Lima et al. (2023) with new locations from this study added. The inset map at the top right shows the distribution range of *O. insularis* from O’Brien et al. (2021) and this study.

All animals were collected off the coast of the Florida Keys, Florida between January 8 and 15, 2021 (Figure 1) by Dynasty Marine. Octopuses were caught in shallow waters (6–7.5 m depth) on light-colored bottom substrates of patch reefs, locally referred to as “whip patches,” i.e., thin strips of limestone and coral reef with small aggregations of vertebrates and invertebrates. Octopus dens were in areas with rocks of ∼30 cm heights and gorgonians on the bottom. These individuals were acquired and maintained for behavioral studies. Species identification using genomic and genetic information were needed to confirm the study species before reporting on behavioral findings in the laboratory experiments (Ramos et al., 2023).

The three octopuses (specimens A, B, and C) were shipped from the Florida Keys to the Laboratory of Integrative Neuroscience and the Comparative Biosciences Center at The Rockefeller University in Manhattan, New York. Octopuses were housed individually in 120-gallon glass aquariums (Aqueon, 121.9 cm length × 45.7 cm width × 71.1 cm height) with various shelters and sources of enrichment. Each tank was connected to a closed circulation system for filtration centered in a 36 gallon three-partition sump (91.4 cm length × 34.6 cm width × 38.1 cm height) (Trigger 36 Crystal Sump) housed underneath the aquarium. Water temperature was regulated with a heating element (Finnex Titanium) and a digital temperature controller (Aqua Logic). Water quality was rigorously monitored and maintained. Waste was extracted from filter socks and cleaned daily. The system also regulated the automated 12 h/12 h light/dark cycle of the overhead light fixtures to simulate natural light conditions. Lighting consisted of LED light bulbs in conical aluminum light fixtures, two 250-W bulbs provided white light in the daytime (07:00–19:30 h) and two 36-W bulbs provided deep red light (660 nm) at night (19:30–07:30 h). Water quality parameters were manually tested multiple times daily for pH, salinity, water temperature, and the concentration of ammonia, nitrates, nitrates using API testing kits and handheld electronic sensors (Hanna Instruments, Woonsocket, RI) to maintain optimal levels for the animal (pH: 8.2–8.5; salinity: 32–34 ppt; water temperature: 23–25 C; ammonia: 0 ppm; nitrates: <20 ppm; nitrites: 0 ppm). The sand was cleaned daily with a siphoning gravel washer to remove organic material including sucker cuticles shed by the octopus, animal excrement, and other small organic material left from food remains and uneaten food. Each octopus was fed 50–150 g pieces of thawed frozen shrimp or whole shrimp once to twice a day (11:00 and 16:00 h). Animals were housed for up to 4 months and died of natural death without any indication of distress or illness. After postmortem inspections of the bodies, they were stored at −20°C. A 0.5-g tissue punch was taken from the arm of specimen A and B, while 0.5 g of both kidney and gill tissue were extracted from specimen C. Samples were kept frozen at −80°C until DNA extraction.

### 2.2 Visual characteristics of *Octopus insularis*

**FIGURE 2:**
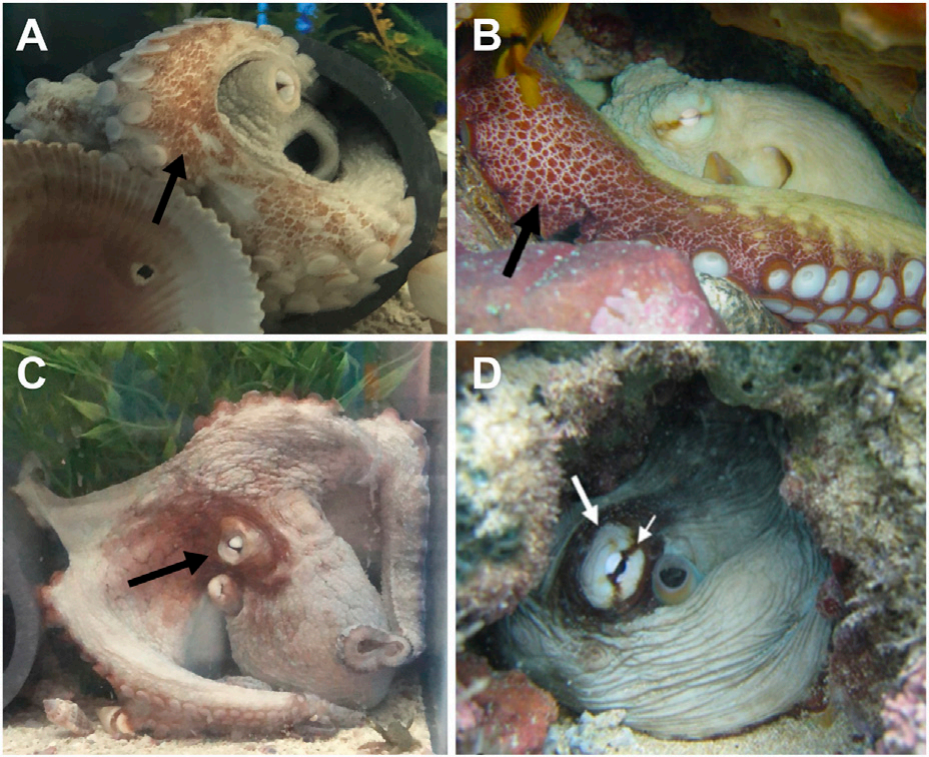
Images of different behaviors and body patterns observed in captive *Octopus insularis* species studied here **(A,C)** and images of the species identified through imagery from O’Brien et al. (2021), images **(B,D)**. All three individuals displayed species-specific patterning for *O. insularis* on the ventral surface of their arms. Deimatic display was confirmed in two animals. **(A)** The red-white reticulated pattern on the ventral surfaces of the arms appeared as patches on a light background and were primarily visible when the animal was within its den. **(C)** Octopus in deimatic display with a white eye, encircled by a light ring, and darkening of the head around the eye. This individual did not regularly present a distinct dark eye bar unlike the wild *O. insularis* **(B,D)**.

Digital video and imagery were used to characterize the physical appearance of the three *O. insularis*. We intermittently filmed the live octopuses with a Canon EOS 80D digital SLR camera fitted with a 55–150 mm lens (1,080 p, 30 fps) or a Sony FDR-AX53 4K Ultra HD Handycam Camcorder (2,160 p, 60 fps). Cameras were mounted on a tripod and positioned <1 m from the tank at a height of 1.5 m, oriented directly at the stationary animal. Imagery was reviewed to identify species-specific body patterns and components enabling confirmation of *O. insularis* (O’Brien et al.*,* 2021). Two distinguishing features that were used to identify *O. insularis* from *O. americanus* (Figures 2A–D): 1) the coloration and patterning of the ventral arm surface described as dark red to purple to brown patches or “red/white reticulate” (Leite and Mather, 2008; Figures 2A, C) and 2) the deimatic display in each species. The deimatic display is found in different octopus species with species-specific characteristics (Packard and Sanders, 1971; Leite and Mather, 2008). In *O. insularis*, the deimatic display consists of a white eye, a pale ring encircling the eye, a dark eye bar, and a broader darkened area around the eye (Leite and Mather, 2008).

### 2.3 *De novo* genome assemblies for genetic species confirmation

Postmortem, all specimens were stored intact at −20°C for use in future studies. For DNA extraction, a 0.5 g tissue punch was taken from the arm of specimen A and B respectively, while 0.5 g of both kidney and gill tissue were extracted from specimen C. Tissue samples were kept frozen at −80°C until further processing. High molecular weight DNA was extracted from each tissue sample using the MagAttract system from Qiagen, which uses a silicon based magnetic bead technology to minimize shearing. Extracted DNA from each animal was then sequenced using the Illumina TruSeq DNA PCR Free kit on a NovaSeq6000. For each specimen, reads were assembled on the Galaxy Project Server, a community-driven web-based analysis platform (Afgan et al., 2018). Reads were trimmed and cleaned prior to assembly using trimmomatic to remove Illumina adapter sequences prior to assembly (Bolger et al., 2014). Raw reads were then checked prior to assembly using the NCBI Foreign Contamination Screen (FCS, 2023) tool (NCBI, 2022). Paired reads from each specimen were then assembled using the St Petersburg genome Assembler (SPAdes) algorithm version 3.9. SPAdes utilizes a de Bruijn graph methodology to optimize the assembly of Illumina short reads (Bankevich et al., 2012; Prjibelski et al., 2020).

### 2.4 Genetic barcode comparison

Expanding on previous work in octopus genetics, we employed a DNA barcoding protocol to compare the sequences of Cytochrome c oxidase subunit I (COI), Cytochrome c oxidase subunit III (COIII), and 16s ribosomal RNA gene (16s). These standard mitochondrial barcode regions have been widely used for species differentiation in octopods (Sӧller et al., 2000; Warnke et al., 2002; Warnke et al., 2004; Warnke et al., 2004; Leite et al., 2008; Guerra et al., 2010; Allcock et al., 2011; Amor et al., 2016; Avendaño et al., 2020b). To identify these regions in each whole-genome assembly, we locally aligned the sequence of *Sepia apama* (Hotaling et al., 2021), an outgroup species to the order Octopoda, to each assembly using the NCBI Blast + algorithm 2.13 (Altschul et al., 1990; Madden and Comacho, 2008). These sequences were then aligned to annotated sequences within Octopoda in the NCBI nucleotide database. For species with annotated sequences from multiple distinct geographic locations, we included at least two distinct sequences for each gene. To determine percent sequence identity, we aligned all available sequences across octopods to that of *S. apama*, as well as our three experimental specimens, using the Multiple Alignment Fast Fourier Transformation (MAFFT) v7.490 (Katoh et al., 2002; Katoh and Standley, 2013) to calculate phylogenetic relation ad pairwise sequence identity. From this alignment, we generated a phylogenetic tree using a Tamura-Nei neighbor-joining model to calculate the distance between sequences based on their percent shared identity (Tamura et al., 2004). We implemented this model through the PhyML 3.3.20180621 phylogenetic tree-generating program (Guindon et al., 2010).

## 3 Results

### 3.1 Visual observations

Similar to previous descriptions of *O. insularis* (Leite et al., 2008; Leite and Mather, 2008; O’Brien et al., 2021), all three octopuses in this study exhibited distinct configurations of patches ranging from dark red to purple to brown on their arms. This feature is described as a “red/white reticulate on ventral arms” (Leite and Mather, 2008; Figures 2A, C). The red/white reticulated pattern on the ventral arm surface was easily identifiable when the animal was resting or sitting in its den with its anterior arm pairs exposed. Two animals were observed displaying a deimatic behavior, presenting a white eye encircled by a light ring within a darkened area on the octopus’s head (Figure 2B). In contrast to previous reports in wild *O. insularis*, individuals in this study did not consistently display a dark eye bar (Figure 2D; O’Brien et al., 2021).

### 3.2 Assembly quality assessment

Across the independent *de novo* assemblies of each specimen, there was a consensus in general statistics, which were generated using gfaststats version 1.3.6 (Formenti et al., 2022). The specimen assemblies were of comparable quality to the currently available reference genome for *O. vulgaris* (Table 1). Among the *de novo* assemblies presented in this study, specimen B generally exhibited the highest quality. It had the fewest contigs in the assembly of the three specimens, despite having the greatest total length, indicating that it is the most intact of the three. Furthermore, the N50, a weighted mean statistic such that 50% of the assembly is composed of reads this length or greater, is highest in specimen B, while the L50, the minimum number of contigs whose length sum 50% of the total assembly size, is lowest in this sample. Across all specimens, the GC% content is approximately 36%, indicating agreement in sequence composition between the distinct assemblies.

**TABLE 1:** Assembly statistics of lab specimen *de novo* genome assemblies and the current *Octopus vulgaris* reference genome (*Octopus vulgaris* (ID 12157)—Genome—NCBI, n.d.).

	Specimen A	Specimen B	Specimen C	*O. vulgaris*
Total length	1,637,756,730	1,622,104,405	1,617,965,393	1,772,957,336
# Contigs	777,909	755,111	845,134	786,906
N50	2,537	2,624	2,178	3,040
L50	181,381	168,259	207,630	137,635
GC%	36.18	36.07	36.22	36.79
Reference Free?	Yes	Yes	Yes	No

**FIGURE 3:**
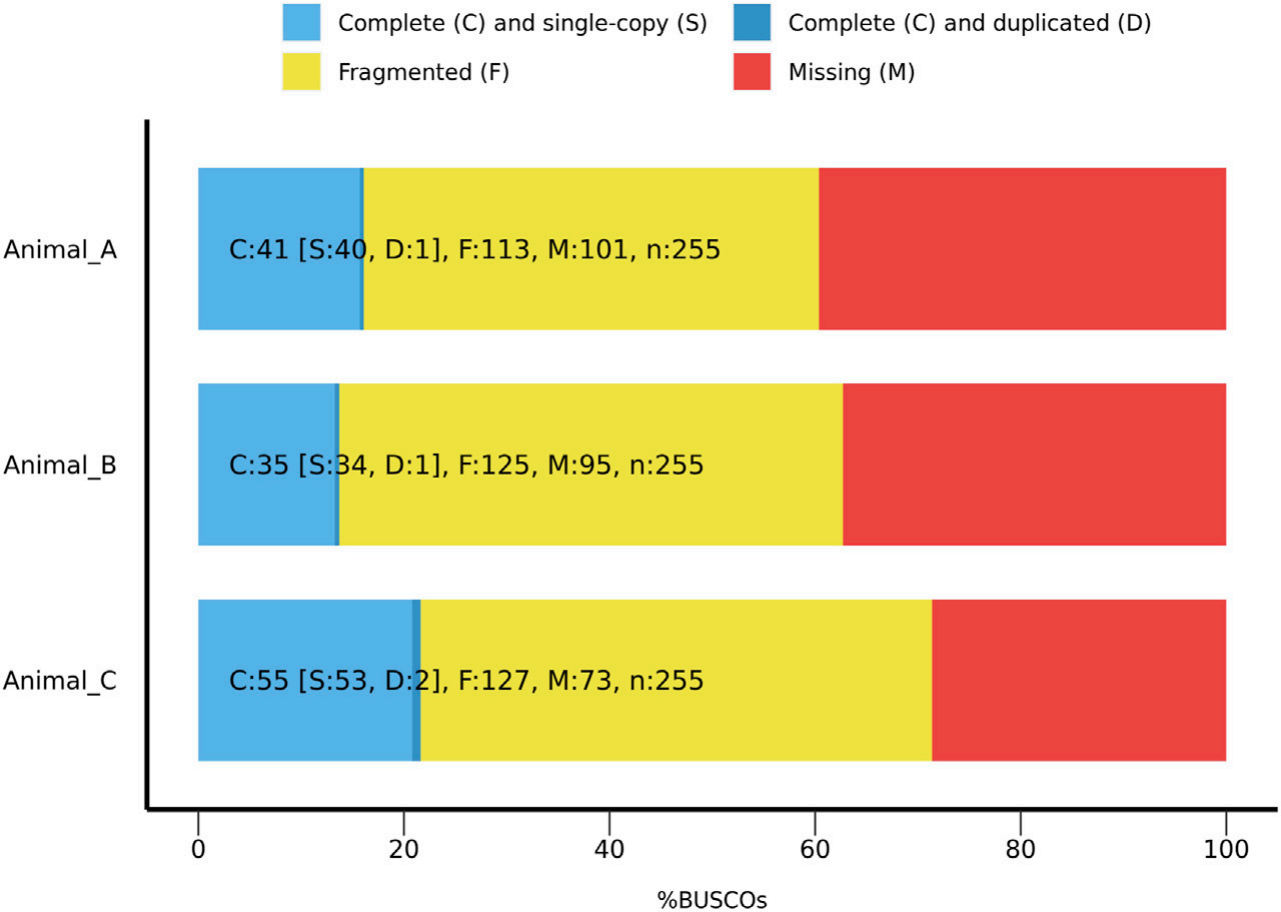
BUSCO ratios for complete, fragmented, and missing orthologs from the eukaryota database 10 across all animals sequenced for 255 orthologous genes, for all three specimens.

To assess accuracy and intactness, these assemblies were screened using the Benchmarking Universal Single-Copy Orthologue (BUSCO) tool version 5.4.6. (Simão et al., 2015). Using the eukaryota ortholog database version 10, each assembly was screened for a set of orthologous genes shared across eukaryotes. Of the 255 orthologs screened, between 13%–22% were found to be intact in each assembly, with 44%–50% of orthologs present in a fragmented form (Figure 3). Raw reads for each specimen had 21-mer histograms generated with Meryl Version 1.3, a genomic kmer counter which assesses the frequency for all reads of a given length *k* (Rhie et al., 2020). To estimate size of the final assembly from the raw data, 21-mer frequency histograms were generated using Genomescope 2.0 (Vurture et al., 2017). From these histograms, Phred score, a measure of consistency of raw read kmer frequency in final assembly, was generated using Merqury version 1.1 (Rhie et al., 2020). All assemblies had a Phred score (QV) greater than 20, indicating a greater than 99% accuracy in assembly of raw reads (Supplementary Table S4; Supplementary Figure S5).

### 3.3 Data availability

All assemblies have been developed under the NCBI BioProject ID PRJNA938087. Currently, assemblies for Specimen A and B are under review. Specimen C, the first to be sequenced, has passed review and been released into GenBank and is publicly available under accession number JARUKP000000000 (https://www.ncbi.nlm.nih.gov/nuccore/JARUKP000000000).

### 3.4 Barcode region comparisons

**FIGURE 4:**
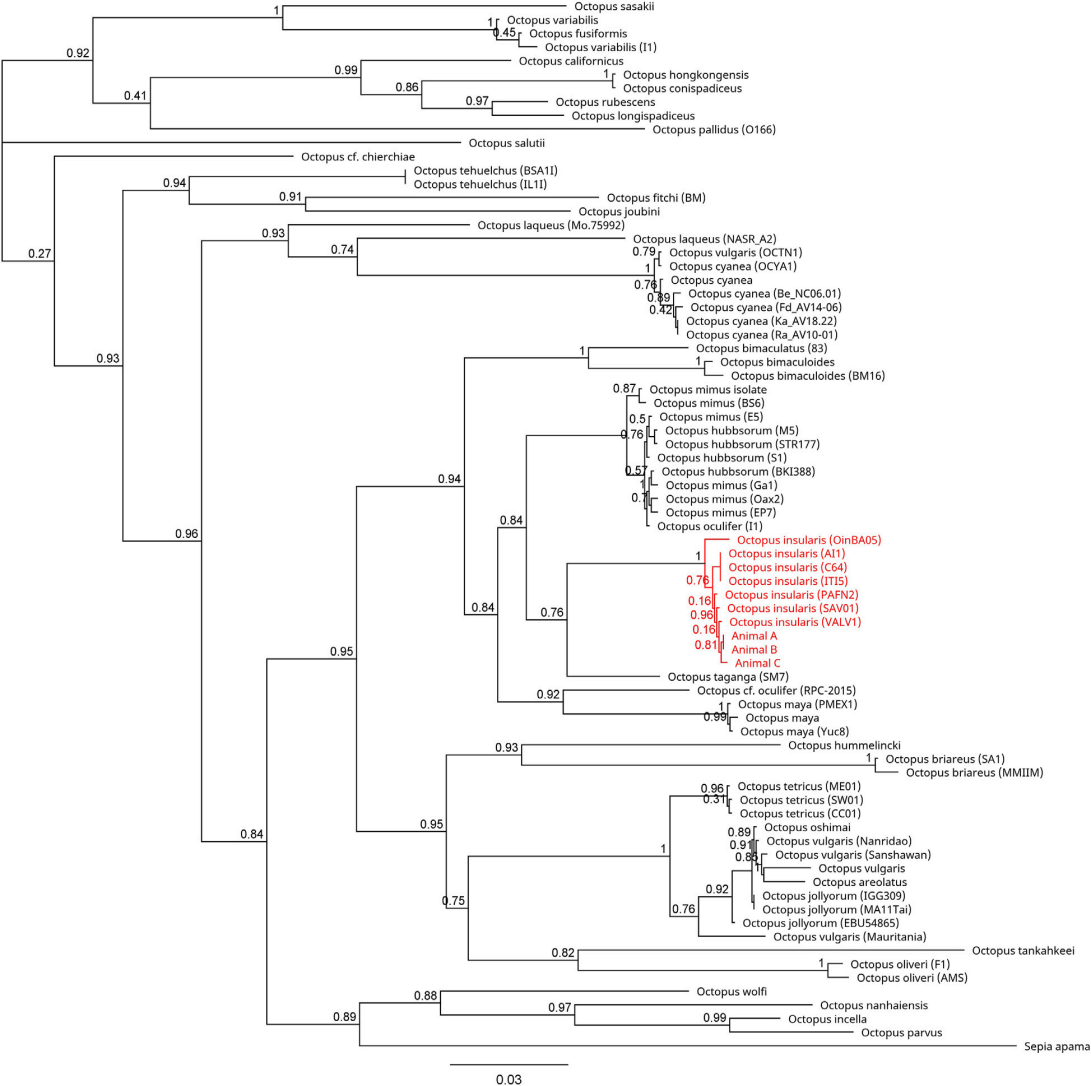
Phylogenetic tree of 77 available COI sequences in octopods and the outgroup *Sepia apama*. Branch length is scaled to indicate the average number of nucleotide substitutions per site. The node capturing the laboratory specimens and *Octopus insularis* is highlighted in red text.

**FIGURE 5:**
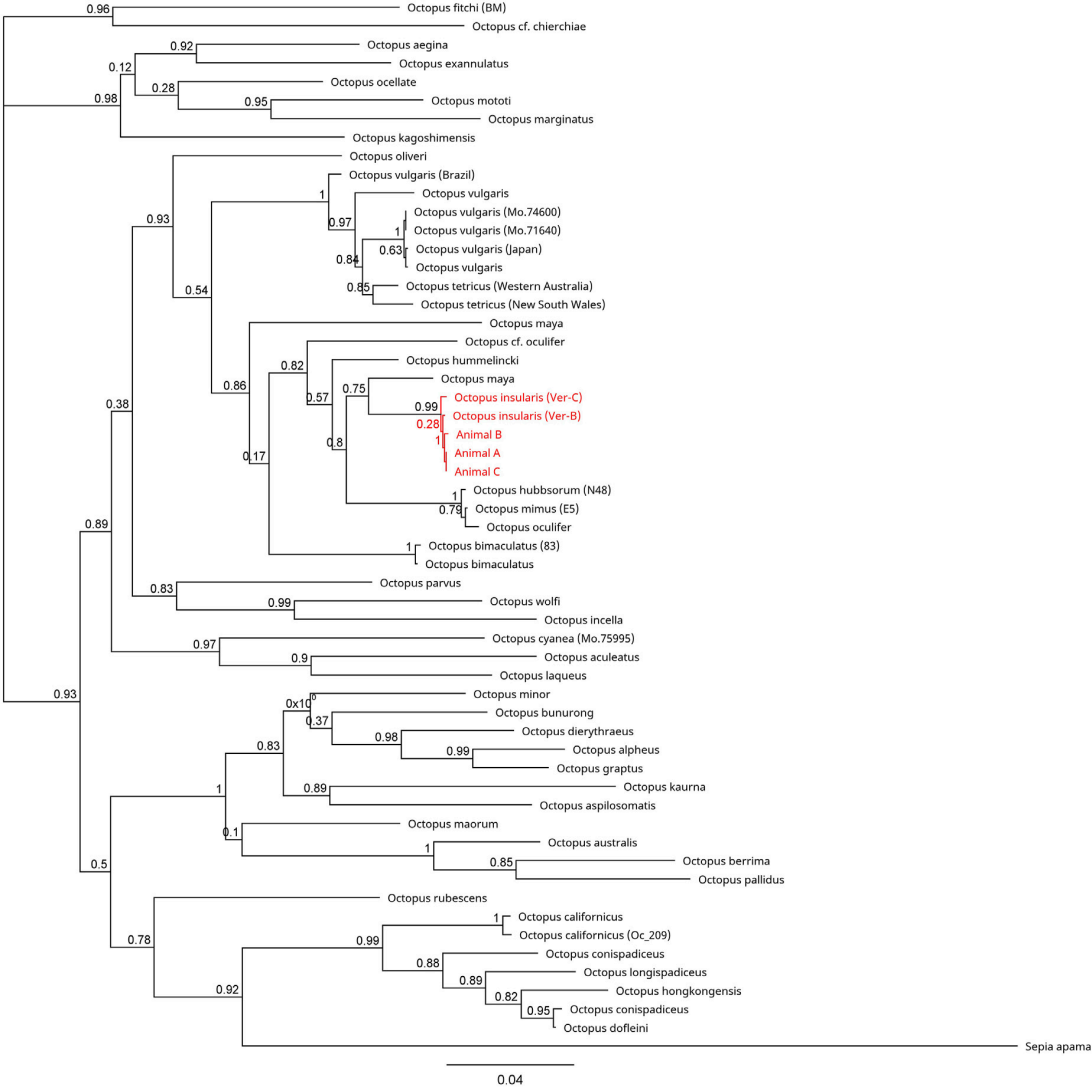
Phylogenetic tree of 57 available COIII sequences in octopods and the outgroup *Sepia apama*. Branch length is scaled to indicate the average number of nucleotide substitutions per site. The node capturing the laboratory specimens and *Octopus insularis* is highlighted in red text.

**FIGURE 6:**
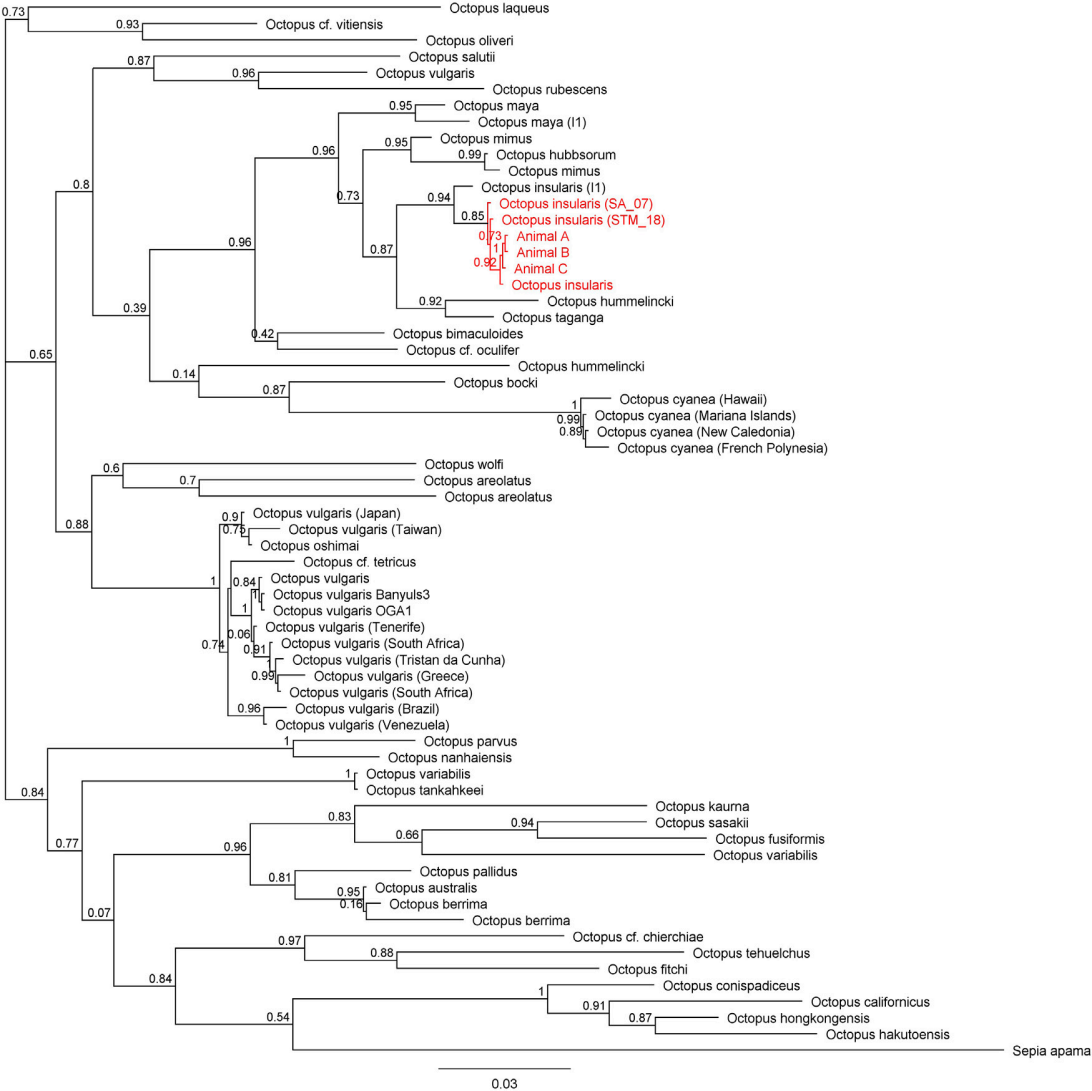
Phylogenetic tree of 65 available 16S sequences in octopods and the outgroup *Sepia apama*. Branch length is scaled to indicate the average number of nucleotide substitutions per site. The node capturing the laboratory specimens and *Octopus insularis* is highlighted in red text.

Across all mitochondrial regions compared, COI sequences exhibited the highest degree of genetic conservation, sharing 84.8% pairwise identity and 32.0% identical sites across 75 specimens representing 49 unique species (Figure 4; Supplementary Table S1). In contrast, available COIII sequences displayed 66.1% pairwise identity and only 1.5% identical sites across 57 sequences from 40 distinct species (Figure 5; Supplementary Table S2). The 16S region showed 78.8% pairwise identity with 10.0% identical sites across 65 specimens, consisting of individuals from 42 distinct species (Figure 6; Supplementary Table S3). In all three mitochondrial barcode regions, laboratory specimens A, B, and C clustered with each other. All three specimens shared highly similar sequences, with only a single nucleotide variation present between specimen C as compared to specimens A and B, which were identical to each other. For all mitochondrial barcodes assessed, the laboratory specimens formed a single clade that exclusively shared a node with the available partial CDS of *O. insularis*.

## 4 Discussion

Here we report the first record of full genomic sequencing for *Octopus insularis* and confirmation of its presence in South Florida. Three specimens were collected from the shallow tropical waters of the Florida Keys and visually identified as *O. insularis* based on distinguishing characteristics of this species (Leite et al., 2008; Leite and Mather, 2008; O’Brien et al., 2021). All mitochondrial barcodes (COI, COIII, 16S) clustered to a monophyletic group with *O. insularis* specimens, supporting evidence of this species in Florida.

*O. insularis* has been described as a generalist, living at a range of depths, temperatures, salinities, habitat types, and now newly proposed geographic areas. O’Brien et al. (2021) first described the sightings of O. *insulari*s in its newly proposed western Atlantic northern range that was recently used as occurrence data for ecological niche models and dispersal simulations in conjunction with molecular methods (*Octopus* spp. collected from Africa) to determine possible transport and dispersal routes for *O. insularis*. Models and molecular results suggest that the distribution of *O. insularis* in America occurs from Florida to Brazil with potential suitable regions throughout the Gulf of Mexico and the Caribbean and trans-Atlantic distribution to Africa (Lima et al., 2023). Our study genetically confirms that the occurrence data for building such models is appropriate for the dispersion of *O. insularis* to Florida. Since these reports, additional sightings of this species have been documented in South Florida (C. O. Bennice, pers. observ).

Although now proposed as a widely dispersed cryptic species, morphological traits and body pattern features have only been described in detail for *O. insularis* in Brazil (Leite et al., 2008; Leite and Mather, 2008). Despite estimates of divergence between *O. insularis* and members of the *O. vulgaris* complex (19-41 million years) morphology of these taxa is relatively conservative except where closely related species occur in sympatry and ecological character displacement may be used as a strategy to reduce resource overlap and facilitate species coexistence (Brown and Wilson, 1956; Amor et al., 2016). However, these sympatric cryptic species in Brazil (*O. vulgaris* and *O. insularis*) have maintained very similar morphology and it is now reported that they coexist in Florida where previous reports of high-density species coexistence occurs (Bennice et al., 2019; Bennice et al., 2021). Morphological traits have been used to successfully distinguish greater species-level diversity within the *O. vulgaris* species complex; however, broad sampling across known distributions to ensure robust morphological analyses has been suggested (Amor et al., 2016). Since both species are generalists, they may compromise and partition resources with little character displacement; however, given *O. insularis* recent geographic expansion and sympatry with many closely related species still warrants investigation of morphological and behavioral traits to ensure species are not misidentified when genetic tools may not be possible or in addition to genetic analysis.

For decades, *O. vulgaris*-like species have been and continue to be inaccurately classified under the species name *O. vulgaris*. Cryptic species are common among octopuses, which may possess subtle or indistinguishable morphological, behavioral, and color pattern traits. Moreover, these distinguishing features may not be adequately captured in video or photo or could be distorted during specimen preservation. We advocate for the inclusion of multiple species recognition mechanisms, encompassing genetic barcoding methods and full genomic sequencing. Numerous mitochondrial barcodes have been established for identifying octopus species, but discrepancies between mitochondrial barcodes still exist (Avendaño et al., 2020a). Additional genes, particularly nuclear genes, have received limited attention and warrant further investigation to determine their efficacy as molecular tools for identification, diversity, and divergence assessment (Amor et al., 2019; Avendaño et al., 2020a). While barcodes enable the comparison of a single region across species of interest, full genome assembly offers the same capability to compare barcodes across species, maximizing the genetic information that can be extracted from such valuable samples. As sequencing technologies become more accessible and cost-effective, whole genome approaches will enable researchers to better characterize variation within cryptic species and their closest relatives.

There is a crucial need for accurate baseline studies to comprehend the distribution, resource utilization, and population biology of cryptic species (Lima et al., 2017; Ángeles-González et al., 2021). The Food and Agriculture Organization (FAO) reports that approximately 100 octopus species are harvested; however, global statistics only identify four (*O. vulgaris*, *Octopus maya*, *Eledone cirrhosa*, and *E. moschata*), with the remaining likely categorized as unidentified octopuses. This study focuses on regions where artisanal fisheries play a significant role, but its findings also have wider implications for the importance of precise species identification in global octopus fisheries. These fisheries are valued at an estimated US$1.07 billion for exports and US$1.33 billion for imports, surpassing many finfish fisheries (Norman and Finn, 2016). Understanding how species baselines change due to environmental pressures, such as climate change, is vital for determining distribution and population connectivity. Genetic analyses are essential for gaining deeper insights into the ecosystem roles and effective fisheries management for these species (Bickford et al., 2007; Lima et al., 2017; Sauer et al., 2020).

## Data Availability

The datasets presented in this study can be found in online repositories. The names of the repository/repositories and accession number(s) can be found below: https://www.ncbi.nlm.nih.gov/bioproject/PRJNA938087.

## Supplementary material

SUPPLEMENTARY FIGURE S5 Merqury table of kmer multiplicity in raw reads and across the assemblies of our samples. Merqury copy number spectrum plots for assemblies of laboratory specimens. Gray regions represent all 21-mers present only in the raw reads and red regions represent all kmers detected in the assembly. **(A)** Spectra ASM ST for Specimen A, **(B)** Spectra ASM ST for Specimen B, and **(C)** Spectra ASM ST for Specimen C.

SUPPLEMENTARY TABLE S1 Shared sequence identity across all COI sequences compared in this study.

SUPPLEMENTARY TABLE S2 Shared sequence identity across all COIII sequences compared in this study.

SUPPLEMENTARY TABLE S3 Shared sequence identity across all 16S sequences compared in this study.

SUPPLEMENTARY TABLE S4 Kmer based statistics of 21 base pair sequences in final assemblies.

## References

[B1] Afgan E. Baker D. Batut B. Van Den Beek M. Bouvier D. Čech M. (2018). The Galaxy platform for accessible, reproducible and collaborative biomedical analyses: 2018 update. Nucleic Acids Res. 46 (1), W537–W544. 10.1093/nar/gky379 29790989 PMC6030816

[B2] Albertin C. B. Simakov O. Mitros T. Wang Z. Y. Pungor J. R. Edsinger-Gonzales E. (2015). The octopus genome and the evolution of cephalopod neural and morphological novelties. Nature 524 (7564), 220–224. 10.1038/nature14668 26268193 PMC4795812

[B3] Allcock A. L. Barratt I. Eleaume M. Linse K. Norman M. D. Smith P. J. (2011). Cryptic speciation and the circumpolarity debate: A case study on endemic southern ocean octopuses using the COI barcode of life. Deep Sea Res. Part II Top. Stud. Oceanogr. 58 (1-2), 242–249. 10.1016/j.dsr2.2010.05.016

[B4] Altschul S. F. Gish W. Miller W. Myers E. W. Lipman D. J. (1990). Basic local alignment search tool. J. Mol. Biol. 215, 403–410. 10.1016/S0022-2836(05)80360-2 2231712

[B5] Ambrose R. F. (1988). Population dynamics of *Octopus bimaculatus*: Influence of life history patterns, synchronous reproduction and recruitment. Malacologia 29 (1), 23–39.

[B6] Amor M. D. Doyle S. R. Norman M. D. Roura A. Hall N. E. Robinson A. J. (2019). Genome-wide sequencing uncovers cryptic diversity and mito-nuclear discordance in the *Octopus vulgaris* species complex. BioRxiv, 573493. 10.1101/573493

[B7] Amor M. D. Laptikhovsky V. Norman M. D. Strugnell J. M. (2017). Genetic evidence extends the known distribution of *Octopus insularis* to the mid-Atlantic islands Ascension and St Helena. J. Mar. Biol. Assoc. U. K. 97, 753–758. 10.1017/S0025315415000958

[B8] Amor M. D. Norman M. D. Roura A. Leite T. S. Gleadall I. G. Reid A. (2016). Morphological assessment of the *Octopus vulgaris* species complex evaluated in light of molecular-based phylogenetic inferences. Zool. Scr. 46, 275–288. 10.1111/zsc.12207

[B9] Anderson R. C. Wood J. B. Mather J. A. (2008). *Octopus vulgaris* in the Caribbean is a specializing generalist. Mar. Ecol. Prog. Ser. 371, 199–202. 10.3354/meps07649

[B11] Ángeles-González L. E. Martínez-Meyer E. Yañez-Arenas C. Velázquez-Abunader I. López-Rocha J. A. Torrejón-Magallanes J. (2021). Climate change effect on *Octopus maya* (voss and solís-ramírez, 1966) suitability and distribution in the yucatan peninsula, Gulf of Mexico: A correlative and mechanistic approach. Estuar. Coast. Shelf Sci. 260, 107502. 10.1016/j.ecss.2021.107502

[B12] Aronson R. B. (1989). The ecology of *Octopus briareus* Robson in a Bahamian saltwater lake. Am. Malacol. Bull. 7, 47–56.

[B13] Avendaño O. Hernández-Flores A. Velázquez-Abunader I. Fernández-Jardón C. Cuevas-Jimenez A. Guerra Á. (2020b). Potential biomass and distribution of octopus in the eastern part of the Campeche Bank (Yucatán, Mexico). Sci. Mar. 84 (2), 1–10. 10.3989/scimar.05007.01A

[B14] Avendaño O. Roura Á. Cedillo-Robles C. E. González Á. F. Rodríguez-Canul R. Velázquez-Abunader I. (2020a). *Octopus americanus*: A cryptic species of the *O. vulgaris* species complex redescribed from the caribbean. Aquat. Ecol. 54, 909–925. 10.1007/s10452-020-09778-6

[B15] Baker M. (2012). De novo genome assembly: What every biologist should know. Nat. Methods 9 (4), 333–337. 10.1038/nmeth.1935

[B16] Bankevich A. Nurk S. Antipov D. Gurevich A. A. Dvorkin M. Kulikov A. S. (2012). SPAdes: A new genome assembly algorithm and its applications to single-cell sequencing. J. Comput. Biol. 19 (5), 455–477. 10.1089/cmb.2012.0021 22506599 PMC3342519

[B17] Bennice C. O. Brooks W. R. Hanlon R. T. (2021). Behavioral Dynamics provide insight into resource exploitation and habitat coexistence of two octopus species in a shallow Florida lagoon. J. Exp. Mar. Biol. Ecol. 151592, 151592–152543. 10.1016/j.jembe.2021.151592

[B18] Bennice C. O. Rayburn A. P. Brooks W. R. Hanlon R. T. (2019). Fine-scale habitat partitioning facilitates sympatry between two octopus species in a shallow Florida lagoon. Mar. Ecol. Prog. Ser. 609, 151–161. 10.3354/meps12845

[B19] Bickford D. Lohman D. J. Sodhi N. S. Ng P. K. Meier R. Winker K. (2007). Cryptic species as a window on diversity and conservation. Trends Ecol. Evol. 22, 148–155. 10.1016/j.tree.2006.11.004 17129636

[B20] Bolger A. M. Lohse M. Usadel B. (2014). Trimmomatic: A flexible trimmer for Illumina sequence data. Bioinformatics 30 (15), 2114–2120. 10.1093/bioinformatics/btu170 24695404 PMC4103590

[B21] Brown W. L. Wilson E. O. (1956). Character displacement. Syst. Zool. 5, 49–64. 10.2307/2411924

[B22] de Beer C. L. Potts W. M. (2013). Behavioural observations of the common octopus *Octopus vulgaris* in Baia dos Tigres, southern Angola. Afr. J. Mar. Sci. 35, 579–583. 10.2989/1814232x.2013.847496

[B23] FCS (2023). NCBI - national center for biotechnology information/NLM/NIH. Avaliable At: https://github.com/ncbi/fcs .

[B24] Formenti G. Abueg L. Brajuka A. Brajuka N. Gallardo-Alba C. Giani A. (2022). Gfastats: Conversion, evaluation and manipulation of genome sequences using assembly graphs. Bioinformatics 38 (17), 4214–4216. 10.1093/bioinformatics/btac460 35799367 PMC9438950

[B25] Forsythe J. W. Hanlon R. T. (1988). Behavior, body patterning and reproductive biology of *Octopus bimaculoides* from California. Malacologia 29, 41–55.

[B26] Gleadall I. G. (2016). *Octopus sinensis* d ‘orbigny, 1841 (cephalopoda: octopodidae): Valid species name for the commercially valuable east asian common Octopus. Species divers. 21, 31–42. 10.12782/sd.21.1.031

[B27] González-Gómez R. de los Angeles Barriga-Sosa I. Pliego-Cárdenas R. Jiménez-Badillo L. Markaida U. Meiners-Man-dujano C. (2018). An integrative taxonomic approach reveals *Octopus insularis* as the dominant species in the Veracruz Reef System (southwestern Gulf of Mexico). PeerJ 6, e6015. 10.7717/peerj.6015 30564516 PMC6286802

[B28] Guerra A. Roura A. González A. F. Pascual S. Cherel Y. Pérez-Losada M. (2010). Morphological and genetic evidence that *Octopus vulgaris* cuvier, 1797 inhabits Amsterdam and Saint Paul islands (southern Indian ocean). ICES J. Mar. Sci. 67 (7), 1401–1407. 10.1093/icesjms/fsq040

[B29] Guerrero-Kommritz J. Camelo-Guarin S. (2016). Two new octopod species (Mollusca: Cephalopoda) from the southern Caribbean. Mar. Biodivers. 46 (3), 589–602. 10.1007/s12526-015-0406-9

[B30] Guerrero-Kommritz J. Rodriguez-Bermudez A. (2019). Soft-bottom octopods (Cephalopoda: Octopodidae) of the southern Caribbean with the description of a new species of Macrotritopus. Mar. Biodivers. 49, 1197–1215. 10.1007/s12526-018-0903-8

[B31] Guindon S. Dufayard J. F. Lefort V. Anisimova M. Hordijk W. Gascuel O. (2010). New algorithms and methods to estimate maximum-likelihood phylogenies: Assessing the performance of PhyML 3.0. Syst. Biol. 59 (3), 307–321. 10.1093/sysbio/syq010 20525638

[B32] Guzik M. T. Norman M. D. Crozier R. H. (2005). Molecular phylogeny of the benthic shallow-water octopuses (Cephalopoda: Octopodinae). Mol. Phylogenet. Evol. 37 (1), 235–248. 10.1016/j.ympev.2005.05.009 16009571

[B33] Hanlon R. T. Conroy L. A. Forsythe J. W. (2008). Mimicry and foraging behaviour of two tropical sand-flat octopus species off North Sulawesi, Indonesia. Zool. J. Linn. Soc. 93, 23–38. 10.1111/j.1095-8312.2007.00948.x

[B35] Hollenbeck N. Scheel D. Gravley M. C. Sage G. K. Toussaint R. Talbot S. L. (2017). Use of swabs for sampling epithelial cells for molecular genetics analyses in *Enteroctopus* . Am. Malacol. Bull. 35 (2), 145–157. 10.4003/006.035.0207

[B36] Katoh K. Misawa K. Kuma K. I. Miyata T. (2002). Mafft: A novel method for rapid multiple sequence alignment based on fast fourier transform. Nucleic Acids Res. 30 (14), 3059–3066. 10.1093/nar/gkf436 12136088 PMC135756

[B37] Katoh K. Standley D. M. (2013). MAFFT multiple sequence alignment software version 7: Improvements in performance and usability. Mol. Biol. Evol. 30 (4), 772–780. 10.1093/molbev/mst010 23329690 PMC3603318

[B38] Kim B. M. Kang S. Ahn D. H. Jung S. H. Rhee H. Yoo J. S. (2018). The genome of common long-arm octopus Octopus minor. Octopus minor. Gigascience 7 (11), giy119. 10.1093/gigascience/giy119 30256935 PMC6279123

[B39] Knowlton N. (1993). Sibling species in the sea. Annu. Rev. Ecol. Evol. Syst. 24, 189–216. 10.1146/annurev.es.24.110193.001201

[B40] Kress W. J. Erickson D. L. (2008). DNA barcodes: Genes, genomics, and bioinformatics. Proc. Natl. Acad. Sci. U.S.A. 105 (8), 2761–2762. 10.1073/pnas.0800476105 18287050 PMC2268532

[B41] Leite T. S. Haimovici M. Mather J. Oliveira J. L. (2009). Habitat, distribution, and abundance of the commercial octopus (*Octopus insularis*) in a tropical oceanic island, Brazil: Information for management of an artisanal fishery inside a marine protected area. Fish. Res. 98, 85–91. 10.1016/j.fishres.2009.04.001

[B42] Leite T. S. Haimovici M. Molina W. Warnke K. (2008). Morphological and genetic description of *Octopus insularis*, a new cryptic species in the *Octopus vulgaris* complex (Cephalopoda: Octopodidae) from the tropical southwestern Atlantic. J. Molluscan Stud. 74, 63–74. 10.1093/mollus/eym050

[B43] Leite T. S. Mather J. A. (2008). A new approach to octopuses’ body pattern analysis: A framework for taxonomy and behavioral studies. Am. Malacol. Bull. 24, 31–41. 10.4003/0740-2783-24.1.31

[B44] Li F. Bian L. Ge J. Han F. Liu Z. Li X. (2020). Chromosome-level genome assembly of the East Asian common octopus (*Octopus sinensis*) using PacBio sequencing and Hi-C technology. Mol. Ecol. Resour. 20 (6), 1572–1582. 10.1111/1755-0998.13216 32603549

[B45] Lima F. D. Ángeles-González L. E. Leite T. S. Lima S. M. (2020a). Global climate changes over time shape the environmental niche distribution of *Octopus insularis* in the Atlantic Ocean. Mar. Ecol. Prog. Ser. 652, 111–121. 10.3354/meps13486

[B46] Lima F. D. Ángeles-González L. E. Maia H. Leite T. S. Cahuich-López M. Mariño-Tapia I. (2023). Molecular data, ecological niche, and dispersal models reveal a Trans-Atlantic shallow-water octopus species. Prog. Oceanogr. 213, 103019. 10.1016/j.pocean.2023.103019

[B47] Lima F. D. Berbel-Filho W. M. Leite T. S. Rosas C. Lima S. M. (2017). Occurrence of *Octopus insularis* leite and Haimovici, 2008 in the tropical northwestern atlantic and implications of species misidentification to octopus fisheries management. Mar. Biodivers. 47, 723–734. 10.1007/s12526-017-0638-y

[B48] Lima F. D. Strugnell J. M. Leite T. S. Lima S. M. (2020b). A biogeographic framework of octopod species diversification: The role of the isthmus of Panama. PeerJ 8, 86911–e8719. 10.7717/peerj.8691 PMC7104719 32257633

[B49] Madden T. Camacho C. (2008). BLAST+ features. Bethesda: National Center for Biotechnology Information US. Available from: https://www.ncbi.nlm.nih.gov/books/NBK569839/ .

[B52] Norman M. D. Finn J. K. (2016). “Cephalopods of the world. An annotated and illustrated catalogue of cephalopod species known to date,” in Octopods and vampire squids No. 4. Editors Jereb P. Roper C. F. E. Norman M. D. Finn J. K. (United Nations: Food and Agriculture Organization), 9–11.

[B53] Norman M. D. (2003). “ *Octopus vulgaris* species complex,” in Cephalopods: A world guide (Hachenheim: ConchBooks), 262–266.

[B54] O’Brien C. E. Bennice C. O. Leite T. (2021). A field guide to distinguishing *Octopus insularis* and *Octopus americanus* (Octopoda: Octopodidae). Zootaxa 5060 (4), 589–594. 10.11646/zootaxa.5060.4.8 34810646

[B55] Packard A. Sanders G. D. (1971). Body patterns of *Octopus vulgaris* and maturation of the response to disturbance. Anim. Behav. 19, 780–790. 10.1016/S0003-3472(71)80181-1

[B56] Prjibelski A. Antipov D. Meleshko D. Lapidus A. Korobeynikov A. (2020). Using SPAdes de novo assembler. Curr. Protoc. Bioinforma. 70 (1), e102. 10.1002/cpbi.102 32559359

[B57] Ramos E. A. Steinblatt M. Demsey R. Reiss D. Magnasco M. O. (2023). Abnormal behavioral episodes associated with sleep and quiescence in *Octopus insularis*: Possible nightmares in a cephalopod? bioRxiv. 10.1101/2023.05.11.540348

[B59] Rhie A. Walenz B. P. Koren S. Phillippy A. M. (2020). Merqury: Reference-free quality, completeness, and phasing assessment for genome assemblies. Genome Biol. 21 (1), 245–327. 10.1186/s13059-020-02134-9 32928274 PMC7488777

[B60] Ritschard E. A. Guerrero-Kommritz J. Sanchez J. A. (2019). First molecular approach to the octopus fauna from the southern Caribbean. PeerJ 1, e7300. 10.7717/peerj.7300 PMC6673601 31392090

[B61] Rosas-Luis R. Jiménez Badillo M. de L. Montoliu-Elena L. Morillo-Velarde P. S. (2019). Food and feeding habits of *Octopus insularis* in the veracruz reef system national park and confirmation of its presence in the southwest Gulf of Mexico. Mar. Ecol. 40, 12535. 10.1111/maec.12535

[B62] Sales J. B. D. L. Do Rego P. S. Hilsdorf A. W. S. Moreira A. A. Haimovici M. Tomás A. R. (2013). Phylogeographical features of *Octopus vulgaris* and Octopus insularis in the Southeastern Atlantic based on the analysis of mitochondrial markers. J. Shellfish Res. 32 (2), 325–339. 10.2983/035.032.0211

[B63] Santana-Cisneros M. L. Rodríguez-Canul R. Zamora-Briseño J. A. Améndola-Pimenta M. De Silva-Dávila R. Ordóñez-López U. (2021). Morphological and molecular identification of Octopoda (Mollusca: Cephalopoda) paralarvae from the southern Gulf of Mexico. Bull. Mar. Sci. 97 (2), 281–304. 10.5343/bms.2020.0027

[B64] Sauer W. H. Gleadall I. G. Downey-Breedt N. Doubleday Z. Gillespie G. Haimovici M. (2020). World Octopus fisheries. Rev. Fish. Sci. Aquac. 29 (3), 279–429. 10.1080/23308249.2019.1680603

[B65] Simão F. A. Waterhouse R. M. Ioannidis P. Kriventseva E. V. Zdobnov E. M. (2015). BUSCO: Assessing genome assembly and annotation completeness with single-copy orthologs. Bioinformatics 31, 3210–3212. 10.1093/bioinformatics/btv351 26059717

[B66] Söller R. Warnke K. Saint-Paul U. Blohm D. (2000). Sequence divergence of mitochondrial DNA indicates cryptic biodiversity in *Octopus vulgaris* and supports the taxonomic distinctiveness of *Octopus mimus* (Cephalopoda: Octopodidae). Mar. Biol. 136, 29–35. 10.1007/s002270050004

[B67] Tamura K. Nei M. Kumar S. (2004). Prospects for inferring very large phylogenies by using the neighbor-joining method. Proc. Natl. Acad. Sci. U.S.A. 101 (30), 11030–11035. 10.1073/pnas.0404206101 15258291 PMC491989

[B68] Vurture G. W. Sedlazeck F. J. Nattestad M. Underwood C. J. Fang H. Gurtowski J. (2017). GenomeScope: Fast reference-free genome profiling from short reads. Bioinformatics 33 (14), 2202–2204. 10.1093/bioinformatics/btx153 28369201 PMC5870704

[B69] Warnke K. Söller R. Blohm D. Saint-Paul U. (2004). A new look at geographic and phylogenetic relationships within the species group surrounding *Octopus vulgaris* (Mollusca, cephalopoda): Indications of very wide distribution from mitochondrial DNA sequences. J. Zool. Syst. Evol. Res. 42, 306–312. 10.1111/j.1439-0469.2004.00277.x

[B70] Warnke K. Söller R. Blohm D. Saint-Paul U. (2002). Assessment of the phylogenetic relationship between *Octopus vulgaris* Cuvier, 1797 and *O. mimus* Gould 1852, using mitochondrial 16s rDNA in combination with morphological characters. Abh. Geol. B. A. 57, 401–405.

[B71] Warnke K. Söller R. Blohm D. Saint-Paul U. (2000). Rapid differentiation between *Octopus vulgaris* Cuvier (1797) and *Octopus mimus* Gould (1852), using randomly amplified polymorphic DNA. J. Zool. Syst. Evol. Res. 38 (2), 119–122. 10.1046/j.1439-0469.2000.382134.x

[B72] Waterston R. H. Lander E. S. Sulston J. E. (2002). On the sequencing of the human genome. Proc. Natl. Acad. Sci. 99, 3712–3716. 10.1073/pnas.042692499 11880605 PMC122589

[B73] Wu Z. Fang D. Yang R. Gao F. An X. Zhuo X. (2018). De novo genome assembly of *Oryza granulata* reveals rapid genome expansion and adaptive evolution. Commun. Biol. 1 (1), 84. 10.1038/s42003-018-0089-4 30271965 PMC6123737

[B74] Xu R. Lue Y. Tang Y. Chen Z. Xu C. Zhang X. (2022). DNA barcoding reveals high hidden species diversity of Chinese waters in the cephalopoda. Front. Mar. Sci. 9, 830381. 10.3389/fmars.2022.830381

[B75] Zarrella I. Herten K. Maes G. E. Tai S. Yang M. Seuntjens E. (2019). The survey and reference assisted assembly of the *Octopus vulgaris* genome. Sci. Data 6, 13. 10.1038/s41597-019-0017-6 30931949 PMC6472339

[B76] Hotaling S. Kelley J. L. Frandsen P. B. (2021). Toward a genome sequence for every animal: Where are we now?. Proceedings of the National Academy of Sciences 118(52), e2109019118. 10.1073/pnas.2109019118 PMC8719868 34862323

